# Effects of *de novo* donor-specific Class I and II antibodies on graft outcomes after liver transplantation: A pilot cohort study

**DOI:** 10.1515/biol-2025-1078

**Published:** 2025-03-22

**Authors:** Necip Altundaş, Eda Balkan, Murat Kizilkaya, Nurhak Aksungur, Salih Kara, Elif Demirci, Ercan Korkut, Gürkan Öztürk, Hakan Dursun

**Affiliations:** Department of General Surgery, Atatürk University, 25240, Erzurum, Turkey; Department of Medical Biology, Faculty of Medicine, Atatürk University, 21 Lalapaşa, 25240, Erzurum, Turkey; Department of Pathology, Atatürk University, 25240, Erzurum, Turkey; Department of Internal Medicine, Atatürk University, 25240, Erzurum, Turkey

**Keywords:** liver transplant immunology, donor-specific antibodies, graft rejection mechanisns, chronic allograft dysfunction, molecular biomarkers in transplantation

## Abstract

This study investigates the role of donor-specific antibodies (DSAs) in liver transplantation outcomes, focusing on their effects on liver damage. Ninety-four patients who underwent liver transplantation between 2019 and 2024 at Atatürk University were included. DSA testing was performed using the Luminex QIAGEN LifeCodes method. Patient demographic data, laboratory results, clinical conditions, and biopsy findings were analyzed. Disease-specific analyses were conducted for Wilson’s disease, autoimmune hepatitis, hepatocellular carcinoma (HCC), and hepatitis B virus (HBV). Due to the limited sample size, larger validation studies are needed, and the impact of the COVID-19 pandemic on the data collection process was considered. At the end of 1 year, persistent DSA had no significant effect on liver damage. However, early DSA positivity, particularly persistence and titration, requires further investigation. In Wilson’s disease, two DSA-positive patients (mean fluorescence intensity [MFI] 1,000–1,500) showed no damage. Among autoimmune hepatitis patients, 5 of 19 were DSA positive (MFI 1,700–5,600), with no detected damage. Four HCC patients were DSA positive (MFI 1,300–2,200). Among HBV patients, 12 of 31 were DSA positive, and 5 experienced liver damage. Tacrolimus levels in the third month were statistically associated with bilirubin levels. Prospective studies are needed to further clarify the clinical significance of DSA.

## 1 Introduction

Liver transplantation has become the main treatment method for end-stage liver disease, with better outcomes being achieved in recent years. Therefore, improving the management of long-term complications, especially chronic antibody-mediated rejection (AMR), is the next challenge to overcome to maintain a good quality of life for liver transplant recipients [1].

The liver is recognized as an immune-privileged organ with anergy (lack of immune response) to many antigens and high immune tolerance. The incidence of AMR after liver transplantation is estimated to be about 1%, much lower than with heart (10–30%) and kidney (20–50%) transplantation [2,3]. Over the past decade, however, several research groups have noted that donor-specific antibodies (DSAs) are associated with chronic rejection. DSAs were reported to be linked to increased rates of liver fibrosis in liver transplant patients followed long term. This indicates the effect of AMR on liver allografts [4,5].

The contribution of anti-human leukocyte antigen (HLA) DSAs to organ rejection and allograft loss is now well documented in kidney, heart, lung, and liver transplants. The introduction of solid-phase analysis technology in the last decade revolutionized the detection and characterization of DSAs and made the method clinically feasible, leading to it becoming the standard of care in the pre- and post-transplant periods [6,7].

The Sensitization in Transplantation: Assessment of Risk (STAR) working group called for the clinical evaluation of alloimmune risks before and after transplantation in their 2017 report. Two types of alloimmune risks were identified in this report: alloimmune memory risk and *de novo*/naive response. The report also discussed how to use HLA typing and anti-HLA antibody tests to more precisely assess patient risk, provided recommendations for transplant programs, and outlined appropriate pre- and post-transplant clinical evaluation methods [8].

The 2019 STAR report examined the clinical applicability and limitations of existing tests for the assessment of alloimmune risk and emphasized the importance of tests evaluating anti-HLA antibody characteristics as a clinical diagnostic and research tool, the potential clinical value of tests measuring alloimmune T- and B-cell memory, and the development of HLA molecular mismatch scores as a prognosis tool. It also reported the clinical validity of DSA tests and stated that DSAs that form after transplantation are strongly associated with graft rejection. The group stated that these tests are widely used in pre- and post-transplantation risk assessment and are regarded as an important endpoint in next-generation clinical trials [9,10].

The earlier 2017 STAR report recommended that the evaluation of anti-HLA antibodies be performed using solid-phase assays and include all major HLA Class I and II loci (HLA-A, -B, -C, -DRB1, -DRB3/4/5, -DQA1/DQB1, -DPA1/DPB1) [11,12]. In addition, threshold values for anti-HLA antibody positivity were defined as the range of 1,000–1,500 mean fluorescence intensity (MFI) as universal cut-off values. Preformed DSA was defined as an antibody detected pre-transplant or between 2 weeks and 3 months post-transplant, while *de novo* DSA was defined as a new antibody that appears after 3 months post-transplant. Recent studies have investigated higher-resolution HLA genotyping to improve DSA risk assessment, evaluating whether DSAs occur pre- or post-transplant and the impact of the presence or loss of post-transplant DSA on graft damage and transplant outcomes. This more precise definition of DSA status is important in defining different forms of AMR and their onset time in liver transplant patients [13].

Although the presence of DSAs is generally less harmful in the context of liver transplantation than with other organ transplants such as kidney and heart, they may adversely affect liver graft function in certain circumstances. In particular, high levels of DSAs may increase the risk of post-transplant chronic graft dysfunction and fibrosis [14].

Therefore, DSA assessment in liver transplant recipients is particularly important for patients who have or are at risk of developing graft dysfunction. Although routine DSA screening is not a standard practice for all liver transplant patients, it is recommended for patients in whom graft health may be affected. This screening can be performed during protocol biopsies or based on specific clinical indicators such as changes in graft function [14].

The aim of this study was to comprehensively investigate the presence of DSAs and the potential effects of these antibodies on graft health in patients with various hepatic diseases undergoing liver transplantation. In particular, by investigating the effects of DSAs on complications such as deterioration in graft function, rejection, and fibrosis, we aimed to contribute to the development of strategies to optimize post-transplant patient management.

## 2 Materials and methods

### 2.1 Study population

This study was conducted collaboratively by the Atatürk University Departments of General Surgery (organ transplantation), Internal Medicine, Medical Biology, and Pathology at Atatürk University Yakutiye Hospital. The study included 94 patients diagnosed with primary hepatocellular carcinoma (HCC), alveolar viral hepatitis B (HBV), cryptogenic hepatitis, autoimmune hepatitis, or Wilson disease, as well as their respective liver donors. There were 48 males (54%) and 46 females (49%) in the patient group and 54 males (55%) and 40 females (45%) in the donor group. The mean age of the patients was 45.72 ± 16.99 years. The research protocol was explained to all participants and an informed consent form was signed before the study.

The patient group underwent a comprehensive battery of tests for the diagnosis of liver disease. These tests play a critical role in determining disease type, severity, and management strategies. First, blood tests were done to assess liver function, specifically examining liver damage by measuring aspartate aminotransferase (AST) and alanine aminotransferase (ALT) enzyme levels. Diseases of the bile ducts were assessed using alkaline phosphatase (ALP) and gamma-glutamyl transferase, and bilirubin metabolic capacity was assessed with jaundice with bilirubin tests. In addition, the ability of the liver to produce proteins and clotting factors was evaluated with albumin and prothrombin time tests. Hepatitis virus and autoantibody tests were conducted to evaluate for viral and autoimmune hepatitis. In terms of imaging methods, liver ultrasound was performed to evaluate liver size, anatomical deformities, and fluid accumulations. Detailed images of the liver were also obtained by computed tomography and magnetic resonance imaging to obtain information about tumors and other abnormalities. In addition, the stiffness of the liver was measured by elastography as part of the assessment for diseases such as fibrosis and cirrhosis. Disease type and severity were also confirmed by microscopic examination of percutaneous liver biopsy specimens. Imaging of the bile and pancreatic ducts was conducted with methods such as endoscopic retrograde cholangiopancreatography and endoscopic ultrasonography.

The study included 94 patients who underwent post-transplant DSA measurements. For patients who received transplants from deceased donors, the DSA molecular test was performed pre-transplantation, while those who received transplants from living donors underwent DSA testing only after transplantation. Patients who died after transplantation were not included in the study. In the post-transplant immunosuppression protocol, steroid therapy was tapered over the first 6 months, aiming for complete discontinuation within the first year after transplantation. In the long term, dual immunosuppressive therapy consisting of calcineurin inhibitor and mycophenolate mofetil was continued. Patients with rejection were treated with rituximab and steroid therapy. This treatment strategy aimed to effectively control the immune response of patients and minimize the risk of rejection.

Patients were followed up for an average of 5 years after liver transplantation, and their demographic, clinical, immunological, and histopathological data were obtained from medical records. In the first year after transplantation, retrospective and prospective evaluations were performed based on a protocol including immunosuppression therapy, *de novo* DSA screening, and allograft biopsies. However, variations in immunosuppression therapy and some missing data limit the evaluation of treatment adherence and immunosuppression.

During the study, a carefully planned method was implemented to minimize the effect of confounders. Participants were assigned or matched to groups based on factors such as disease type, age, sex, and other clinical characteristics. This approach aimed to reduce the impact of potential confounding variables. In addition, during data analysis, detailed statistical analyses were performed to accurately evaluate the effects of factors such as liver diseases and transplant history on the groups. The effects of these variables on the results were minimized by taking inequalities into consideration. These measures aimed to minimize the effects of confounders and increase the reliability of the study.


**Informed consent:** Informed consent has been obtained from all individuals included in this study.
**Ethical approval:** The research related to human use has been complied with all the relevant national regulations and institutional policies and in accordance with the tenets of the Helsinki Declaration and has been approved by the Ethics Committee of Atatürk University Faculty of Medicine (approval no: B.30.2.ATA.0.01.00/162; Erzurum, Türkiye).

### 2.2 Pathologic studies: post-transplant liver biopsy evaluation

The reports of post-transplant liver biopsies performed between 2019 and 2024 at the Department of Pathology of Atatürk University Faculty of Medicine were obtained from the archives for re-evaluation. Prepared archive slides consisted of hematoxylin and eosin (H&E)-stained slides, histochemical slides (Masson’s trichome, reticulin, rhodamine, Perls Prussian blue stains), and immunohistochemical slides (C4d, cytokeratin 7, CD68, CD4, CD8). On examination of the H&E slides, portal inflammation, the dominant inflammatory cell type, and lobular inflammation were evaluated and scored using the modified hepatic activity index (HAI) criteria. The H&E and immunohistochemical slides were evaluated together for central venulitis, portal venulitis, bile duct loss in the portal area, age-related findings in bile duct epithelium (epithelial cell hyperchromasia, nucleolus prominence, cellular disorganization) according to the Banff criteria. Additionally, c4D-stained slides were evaluated immunohistochemically for AMR according to the Banff criteria.

### 2.3 Molecular studies: donor-specific antibody (DSA) protocol

This study was designed to investigate the relationships between *de novo* DSA formation and the primary etiology of liver disease, demographic and laboratory results, the impact of *de novo* DSA on graft histology, the effect of immunosuppressive therapy on DSA development in the graft, and the number of biopsy-confirmed rejection events associated with *de novo* DSA.

Between 2019 and 2024, only DSA levels (expressed as MFI values) were used to evaluate immune responses. In this process, HLA antibodies with an MFI value >1,000 were considered positive. Serum samples were obtained within 1 year after transplantation and analyzed using Luminex-based systems according to the manufacturer’s instructions. For deceased donor transplants, the DSA test was performed before transplantation, and additional tests were conducted as clinically necessary.

*De novo* DSA development was defined as the detection of new antibodies with MFI >1,000 at 1 year post-transplantation. DSAs were evaluated separately for major histocompatibility complex (MHC) Classes I and II.

The Qiagen cell isolation and DSA protocol used in this study was meticulously designed to ensure high accuracy and reliability in laboratory analyses. The protocol begins with the collection of blood samples into anticoagulant tubes, followed by centrifugation to separate plasma and cellular fractions. During this step, white blood cells are enriched and purified by removing plasma. The isolated cells are then cultured in the appropriate nutrient medium or cryogenically stored.

Serum or plasma samples prepared for DSA analysis are processed in the Qiagen Fluorescent Labeled Antibody Identification system to detect anti-HLA antibodies. This system is used to determine the presence and concentration of antibodies, with DSAs detected by measuring the MFI values. Throughout the analysis, the accuracy of the results is ensured through the use of standard controls and calibrations. The data obtained are evaluated according to clinical and laboratory standards, and the results are presented as clinical reports and research findings.

In this study, IgG-type antibodies were used for DSA measurements. These tests do not target specific antigens but instead detect antibodies against HLA Class I and Class II antigens. In the Luminex-based system analysis, Class I and Class II antibodies were evaluated separately, and the positivity for both classes was determined by MFI values. The Luminex Single Antigen panel was not used; instead, general antibody levels for Class I and Class II were measured.

DSA tests were performed on the Luminex device using the IMMUCOR DSA kit from Qiagen. The test was conducted in accordance with the protocol provided by the kit, with Class I and Class II antigens being evaluated in a general manner. The tests were analyzed under two main categories without examining each locus individually.

This protocol provides critical information for immunological and transplantation studies by ensuring high sensitivity and reliability in DSA detection and cell isolation. DSA measurements were conducted using IMMUCOR/LIFECODES brand systems (lot number 3014433), which are manufactured in the United States and were used according to the manufacturer’s instructions.

### 2.4 Statistical analysis

The data were analyzed with IBM SPSS Statistics version 23. The normality of data distributions was examined with the Shapiro–Wilk test. Connections between categorical variables were examined with the Fisher–Freeman–Halton test. One-way analysis of variance (ANOVA) was used to compare normally distributed data between three or more groups. The Kruskal–Wallis test was used to compare non-normally distributed data between three or more groups, followed by the Dunn test for multiple comparisons. Associations between independent risk factors and rejection were examined by binary logistic regression analysis. Results were presented as mean ± standard deviation or median and range for quantitative data and as frequency and percentage for categorical data. *p* < 0.05 was accepted as the level of statistical significance.

## 3 Results

A total of 94 patients who underwent their first liver transplantation at Erzurum Atatürk University Organ Transplantation Center between 2019 and 2024 were followed up with *de novo* DSA screening and allograft biopsy in the first year after transplantation. The patients were categorized according to diagnosis, and data regarding demographic characteristics, *de novo* DSA prevalence, risk factors, and allograft histology were presented for each diagnostic group. Demographic data and laboratory results were meticulously collected and information about the patients’ immunosuppressive drug therapy was recorded in detail. However, there were still some missing biopsy data and drug levels. As these missing data may pose a limitation when evaluating the efficacy of immunosuppressive therapies and biopsy results, the findings of this study should be interpreted with caution.

### 3.1 Demographic and transplant characteristics

Between 2019 and 2024, a total of 94 patients in our institution underwent their first liver transplantation from a living or deceased donor. The basic characteristics of the liver transplant recipients are detailed in Table 1. The study included patients diagnosed with primary HCC, alveolar disease, HBV, cryptogenic hepatitis, autoimmune hepatitis, or Wilson disease, as well as their respective liver donors. There were 48 males (54%) and 46 females (49%) in the patient group and 54 males (55%) and 40 females (45%) in the donor group. The mean age of the patients was 45.72 ± 16.99 years.

**Table 1:** Comparison of demographic and clinical data by diagnosis

	Alveolar echinococcosis	HBV	HCC	Cryptogenic hepatitis	Autoimmune hepatitis	Wilson disease	Total	Test statistic	*p*
Age (years)	42.54 ± 16.38	51.25 ± 15.84	46.55 ± 12.11	44.57 ± 14.24	43.05 ± 19.08	30.2 ± 26.01	45.72 ± 16.99	1.788	0.124^a^
Transplant duration (years)	5 (1–10)	6 (0–12)	5 (1–11)	4.5 (1–10)	5 (0–12)	5 (5–10)	5 (0–12)	2.687	0.748^b^
Sex, *n* (%)									
Male	8 (61.5)	14 (43.8)	6 (54.6)	8 (57.1)	10 (52.6)	2 (40)	48 (51.1)	1.914	0.894^c^
Female	5 (38.5)	18 (56.3)	5 (45.5)	6 (42.9)	9 (47.4)	3 (60)	46 (48.9)		
ALP (U/L)	118.9 (73–290)	132 (49.1–391.2)	161.73 (36–290)	88.95 (37.4–342.6)	112.7 (27–196.6)	191.5 (71–303.8)	118.9 (27–391.2)	3.612	0.607^b^
AST (U/L)	22.8 (8.4–39.5)	22.4 (12.8–2,550.1)	30.5 (8.4–39.4)	23.6 (14.6–1,776.2)	23.5 (11.9–2,550.1)	20.9 (15.9–59.8)	22.8 (8.4–2,550.1)	3.385	0.641^b^
ALT (U/L)	21.6 (7–135.7)	25.9 (8.5–2,734.7)	28 (8–58)	24.2 (12.5–692.1)	29.7 (9.9–1,004.7)	20 (11.6–82)	25.9 (7–2,734.7)	5.333	0.377^b^
Total bilirubin (mg/dL)	0.95 (0.41–2.75)	0.91 (0.29–14.51)	0.82 (0.34–2.65)	0.84 (0.43–2.59)	0.84 (0.54–14.51)	0.71 (0.42–1.19)	0.84 (0.29–14.51)	2.351	0.799^b^
Direct bilirubin (mg/dL)	0.23 (0.11–1.24)	0.22 (0.06–9.22)	0.22 (0.07–1.29)	0.2 (0.1–0.88)	0.19 (0.1–9.22)	0.14 (0.11–0.29)	0.21 (0.06–9.22)	4.366	0.498^b^

Values are represented as *n* (%), mean ± standard deviation, and median (range).

^a^One-way ANOVA.

^b^Kruskal–Wallis *H* test.

^c^Fisher–Freeman–Halton test.

Abbreviations: HBV: viral hepatitis B, HCC: hepatocellular carcinoma, ALP: alkaline phosphatase, AST: aspartate transaminase, ALT: alanine transaminase.

There was no statistically significant difference in mean age according to diagnosis (*p* = 0.124). The mean age was 42.54 years in the alveolar disease group, 51.25 years in the HBV group, 46.55 years in the HCC group, 44.57 years in the cryptogenic hepatitis group, 43.05 years in the autoimmune hepatitis group, and 30.2 years in the Wilson disease group. There was no statistically significant difference in median transplant time according to diagnosis (*p* = 0.748). The median time was 4.5 years among patients with cryptogenic hepatitis; 5 years for patients with alveolar disease, HCC, autoimmune hepatitis, and Wilson disease; and 6 years in patients with HBV.

There was no statistically significant difference between the diagnostic groups in terms of median ALP value (*p* = 0.607). The median ALP level was 118.9 in the alveolar disease group, 132 in the HBV group, 161.73 in the HCC group, 88.95 in the cryptogenic hepatitis group, 112.7 in the autoimmune hepatitis group, and 191.5 in the Wilson disease group. There were also no statistically significant differences in median AST or ALT values according to diagnosis (*p* = 0.641 and *p* = 0.377, respectively). The median AST and ALT levels were 22.8 and 21.6 among patients with alveolar disease, 22.4 and 25.9 among those with HBV, 30.5 and 28 among those with HCC, 23.6 and 24.2 for cryptogenic hepatitis, 23.5 and 29.7 for autoimmune hepatitis, and 20.9 and 20 among patients with Wilson disease, respectively. Similarly, median total bilirubin and direct bilirubin levels showed no significant difference among the diagnostic groups (*p* = 0.799 and *p* = 0.498, respectively). The median total and direct bilirubin values were 0.95 and 0.23 in the alveolar disease group, 0.91 and 0.22 in the HBV group, 0.82 and 0.22 in the HCC group, 0.84 and 0.20 in the cryptogenic hepatitis group, 0.84 and 0.19 in the autoimmune hepatitis group, and 0.71 and 0.14 in the Wilson disease group. There was no statistically significant relationship between sex and diagnosis (*p* = 0.894) (Table 1).

#### 3.1.1 De novo DSA Class I and Class II classifications and MFI values of liver transplant patients according to diagnosis

The distribution of *de novo* DSA positivity and MFI values by diagnostic group are presented in Table 2. There were no statistically significant differences between the diagnostic groups in the distribution of Class I (*p* = 0.972), Class II (*p* = 0.901), or Class I and Class II (*p* = 0.934) *de novo* DSA positivity.

**Table 2:** Comparison of Class I and II DSAs and MFI values according to diagnosis

Parameter	Alveolar echinococcosis	HBV	HCC	Cryptogenic hepatitis	Autoimmune hepatitis	Wilson disease	Total	Test statistic	*p*
Class I									
Negative	11 (84.6)	25 (78.1)	10 (90.9)	11 (78.6)	16 (84.2)	4 (80)	77 (81.9)	1.329	0.972^a^
Positive	2 (15.4)	7 (21.9)	1 (9.1)	3 (21.4)	3 (15.8)	1 (20)	17 (18.1)		
Class II									
Negative	9 (69.2)	21 (65.6)	7 (63.6)	9 (64.3)	15 (79)	3 (60)	64 (68.1)	1.807	0.901^a^
Positive	4 (30.8)	11 (34.4)	4 (36.4)	5 (35.7)	4 (21.1)	2 (40)	30 (31.9)		
Classes I and II									
Negative	9 (69.2)	20 (62.5)	7 (63.6)	8 (57.1)	14 (73.7)	3 (60)	61 (64.9)	1.509	0.934^a^
Positive	4 (30.8)	12 (37.5)	4 (36.4)	6 (42.9)	5 (26.3)	2 (40)	33 (35.1)		
Class I MFI	364 (62–1,559)	574 (24–4,383)	443 (134–2,160)	385 (39–4,431)	500 (133–4,498)	457 (287–1,552)	456 (24–4,498)	1.813	0.874^b^
Class II MFI	559 (65–2,913)	652 (60–7,310)	648 (213–1,876)	666.5 (59–5,477)	530 (123–5,514)	564 (479–1,452)	589 (59–7,310)	1.486	0.915^b^

Values are represented as *n* (%), mean ± standard deviation, and median (range).

^a^Fisher–Freeman–Halton test.

^b^Kruskal–Wallis *H* test.

The median MFI values for Class I DSAs did not differ by diagnosis (*p* = 0.874). The median values were 364 for alveolar disease, 574 for HBV, 443 for HCC, 385 for cryptogenic hepatitis, 500 for autoimmune hepatitis, and 457 for Wilson disease. Similarly, there was no statistically significant difference in median MFI values of Class II DSAs according to diagnosis (*p* = 0.915). The median values were 559 for alveolar disease, 652 for HBV, 648 for HCC, 666.5 for cryptogenic hepatitis, 530 for autoimmune hepatitis, and 564 for Wilson disease (Table 2).

#### 3.1.2 Pathological biopsy findings in liver transplant patients according to diagnosis

Rejection status and other biopsy findings are compared between the diagnostic groups in Table 3. Overall, 73.4% of the patients had rejection and 26.6% did not. Differences in the frequency of rejection were observed between the groups, but the difference did not reach statistical significance (*p* = 0.069).

**Table 3:** Comparison of rejection and liver biopsy results according to diagnosis

Parameter	Alveolar echinococcosis	HBV	HCC	Cryptogenic hepatitis	Autoimmune hepatitis	Wilson disease	Total	Test statistic	*p*
Rejection status									
No	11 (84.6)	24 (75)	10 (90.9)	6 (42.9)	13 (68.4)	5 (100)	69 (73.4)	9.756	0.069^a^
Yes	2 (15.4)	8 (25)	1 (9.1)	8 (57.1)	6 (31.6)	0 (0)	25 (26.6)		
Portal inflammation severity									
1	1 (20)	3 (37.5)	0 (0)	1 (16.7)	1 (25)	—	6 (24)	10.380	0.639^a^
2	1 (20)	2 (25)	2 (100)	3 (50)	2 (50)	—	10 (40)		
3	3 (60)	1 (12.5)	0 (0)	2 (33.3)	0 (0)	—	6 (24)		
4	0 (0)	2 (25)	0 (0)	0 (0)	1 (25)	—	3 (12)		
Lobular inflammation									
K1	1 (20)	1 (16.7)	1 (50)	1 (20)	1 (50)	—	5 (25)	7.183	0.556^a^
K2	3 (60)	1 (16.7)	1 (50)	1 (20)	1 (50)	—	7 (35)		
K3	1 (20)	4 (66.7)	0 (0)	3 (60)	0 (0)	—	8 (40)		
Fibrosis									
2	3 (75)	1 (16.7)	0 (0)	1 (20)	1 (50)	—	6 (31.6)	13.207	0.189^a^
3	1 (25)	1 (16.7)	2 (100)	3 (60)	0 (0)	—	7 (36.8)		
4	0 (0)	1 (16.7)	0 (0)	1 (20)	1 (50)	—	3 (15.8)		
5	0 (0)	3 (50)	0 (0)	0 (0)	0 (0)	—	3 (15.8)		
Ductulitis									
No	13 (100)	32 (100)	11 (100)	9 (64.3)	16 (84.2)	5 (100)	86 (91.5)	14.411	0.001^a^
Yes	0 (0)	0 (0)	0 (0)	5 (35.7)	3 (15.8)	0 (0)	8 (8.5)		
Central venulitis									
No	13 (100)	32 (100)	11 (100)	11 (78.6)	17 (89.5)	5 (100)	89 (94.7)	8.239	0.041^a^
Yes	0 (0)	0 (0)	0 (0)	3 (21.4)	2 (10.5)	0 (0)	5 (5.3)		
Portal venulitis									
No	13 (100)	32 (100)	11 (100)	10 (71.4)	16 (84.2)	5 (100)	87 (92.6)	11.635	0.009^a^
Yes	0 (0)	0 (0)	0 (0)	4 (28.6)	3 (15.8)	0 (0)	7 (7.4)		
Bile duct loss									
No	13 (100)	32 (100)	11 (100)	11 (78.6)	19 (100)	5 (100)	91 (96.8)	8.936	0.015^a^
Yes	0 (0)	0 (0)	0 (0)	3 (21.4)	0 (0)	0 (0)	3 (3.2)		
Hyperchromatosis									
No	13 (100)	30 (93.8)	10 (90.9)	9 (64.3)	17 (89.5)	5 (100)	84 (89.4)	8.468	0.068^a^
Yes	0 (0)	2 (6.3)	1 (9.1)	5 (35.7)	2 (10.5)	0 (0)	10 (10.6)		
Nucleolus									
No	13 (100)	30 (93.8)	10 (90.9)	9 (64.3)	17 (89.5)	5 (100)	84 (89.4)	8.468	0.068^a^
Yes	0 (0)	2 (6.3)	1 (9.1)	5 (35.7)	2 (10.5)	0 (0)	10 (10.6)		
Cellular disorganization									
No	13 (100)	30 (93.8)	10 (90.9)	9 (64.3)	17 (89.5)	5 (100)	84 (89.4)	8.468	0.068^a^
Yes	0 (0)	2 (6.3)	1 (9.1)	5 (35.7)	2 (10.5)	0 (0)	10 (10.6)		
C4d									
C4d negative	0 (0)	—	1 (100)	4 (100)	2 (100)	—	7 (87.5)	—	—
C4d positive	1 (100)	—	0 (0)	0 (0)	0 (0)	—	1 (12.5)		
Pathological diagnosis									
Acute rejection	1 (100)	0 (0)	0 (0)	0 (0)	0 (0)	—	1 (6.3)	18.896	0.491^a^
Acute cellular rejection	0 (0)	0 (0)	0 (0)	1 (14.3)	1 (20)	—	2 (12.5)		
Early chronic T-cell-mediated rejection	0 (0)	1 (50)	1 (100)	5 (71.4)	3 (60)	—	10 (62.5)		
Steatohepatitis	0 (0)	1 (50)	0 (0)	0 (0)	1 (20)	—	2 (12.5)		

Values are represented as *n* (%).

^a^Fisher–Freeman–Halton test.

The severity of portal inflammation was rated in four categories. The distribution of patients according to this severity rating (from 1 to 4) was 24, 40, 24, and 12%, respectively. There was no significant difference in portal inflammation severity (*p* = 0.639). Lobular inflammation was rated in three categories, K1, K2, and K3. The distribution of patients in these categories was 25, 35, and 40%, respectively. Lobular inflammation did not differ significantly between the groups (*p* = 0.556). The severity of fibrosis was graded as 2, 3, 4, and 5. According to the severity rating, the distribution of fibrosis was 31.6, 36.8, 15.8, and 15.8%, respectively. Fibrosis severity did not differ significantly (*p* = 0.189) (Table 3).

#### 3.1.3 Immunosuppressant drug serum levels of recipients after liver transplantation

Immunosuppressant drug levels did not differ statistically overall or according to diagnosis (Table 4).

**Table 4:** Immunosuppressant drug levels at 1, 3, and 6 months post-transplant

Drug	Alveolar echinococcosis	HBV	HCC	Cryptogenic hepatitis	Autoimmune hepatitis	Wilson disease	Total	Test statistic	*p*
1-month TAC (UL)	6.37 (3.6–15)	8.37 (3.6–13.2)	7.11 (6.24–19.1)	8.8 (4.5–14.1)	7.19 (2.6–10.76)	7.8 (5.5–13.23)	7.38 (2.6–19.1)	8.145	0.148^a^
3-month TAC (UL)	8.1 (5.83–15.52)	9.15 (5.5–14.8)	8.3 (6.07–12.4)	9.69 (4.65–12.18)	9.2 (1.78–12.67)	8.1 (4.9–9.8)	8.87 (1.78–15.52)	3.324	0.650^a^
6-month TAC (UL)	8.6 ± 2.39	8.69 ± 2.1	8.75 ± 2.24	9.28 ± 1.59	8.54 ± 2.27	7.02 ± 1.76	8.65 ± 2.11	0.857	0.514^b^
Basiliximab (µg/L)	0.30–9.27	0.24–7.40	0.92–6.48	0.23–4.68	0.30–9.20	0.65–8.87	0.21–7.44	0.38–8.00	0.436^b^

Values are represented as mean ± standard deviation and median (range).

^a^Kruskal–Wallis *H* test.

^b^One-way ANOVA.

Abbreviations: HBV: viral hepatitis B, HCC: hepatocellular carcinoma, TAC: tacrolimus.

There was no statistically significant difference in median tacrolimus levels among the diagnostic groups at post-transplant 1 month (*p* = 0.148). The median tacrolimus values at 1 month were 6.37 ng/mL in the alveolar disease group, 8.37 in the HBV group, 7.11 in the HCC group, 8.8 in the cryptogenic hepatitis group, 7.19 in the autoimmune hepatitis group, and 7.8 in the Wilson disease group. Similarly, these values did not differ at post-transplant 3 or 6 months (*p* = 0.650 and 0.514, respectively). At 3 and 6 months, the median tacrolimus values were 8.1 and 8.6 in the alveolar disease group, 9.15 and 8.69 in the HBV group, 8.3 and 8.75 in the HCC group, 9.69 and 9.28 in the cryptogenic hepatitis group, 9.2 and 8.54 in the autoimmune hepatitis group, and 8.1 and 7.02 in the Wilson disease group, respectively. There was also no statistically significant difference in basiliximab levels according to diagnosis (*p* = 0.436) (Table 4).

#### 3.1.4 Demographic, laboratory, and immunosuppressant drug data according to the presence of post-transplant *de novo* DSAs

**Figure 1:**
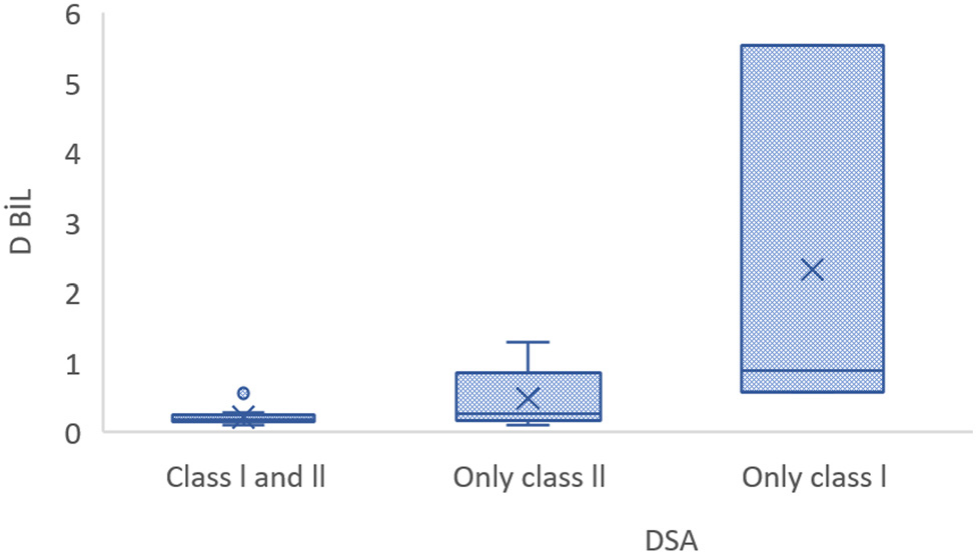
Direct bilirubin levels in patients positive for DSAs against Class I and/or Class II HLA.

**Figure 2:**
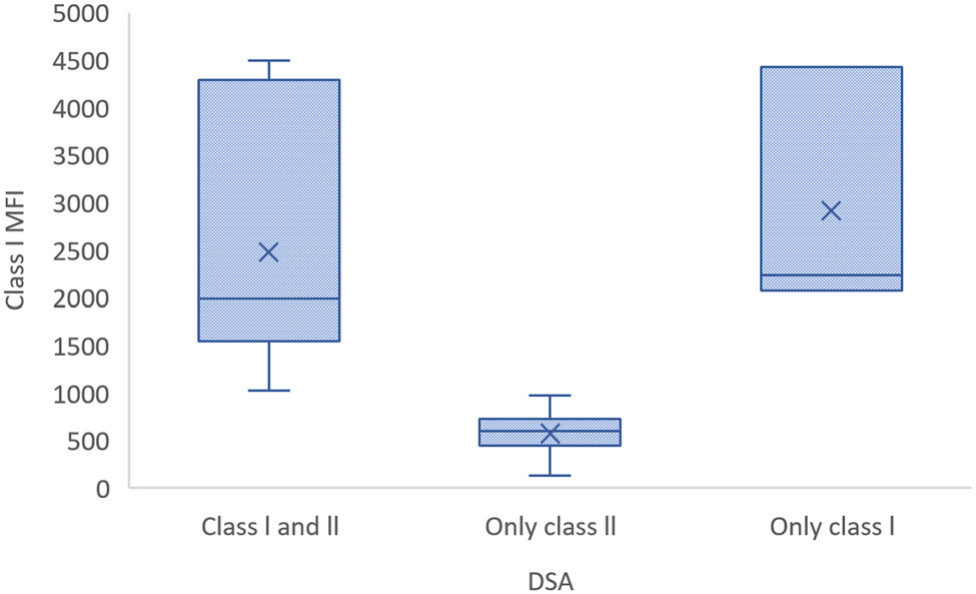
Class I MFI values in patients positive for DSAs against Class I and/or Class II HLA.

**Figure 3:**
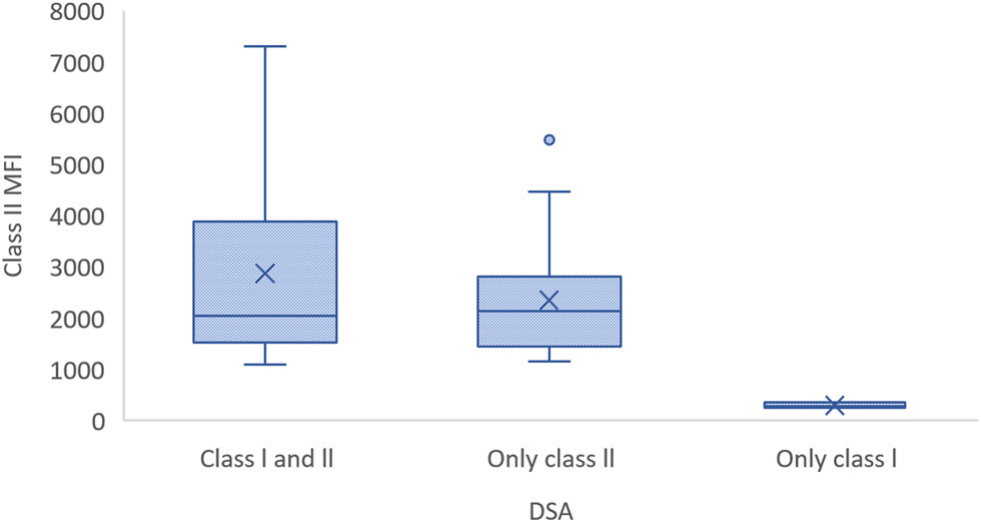
Class II MFI values in patients positive for DSAs against Class I and/or Class II HLA.

There was a statistically significant difference in direct bilirubin levels according to DSA class (*p* = 0.008). The median direct bilirubin levels were 0.88 in patients positive for Class I only, 0.27 in those who were positive for Class II only, and 0.19 in those positive for both Classes I and II. Significant differences were also observed in median MFI values when categorized according to DSA class. The median Class I MFI value showed no significant difference between patients positive for only Class I and those positive for both Classes I and II but was significantly lower in patients positive for Class II only (*p* < 0.001). Similarly, the median Class II MFI value did not differ significantly between patients positive for only Class II and those positive for both Classes I and II but was significantly lower among patients positive for Class I only (*p* = 0.016). No significant difference in the other demographic and clinical variables was observed according to DSA class (*p* > 0.05) (Table 5) (Figures 1–3).

**Table 5:** Comparison of quantitative data according to the presence of donor-specific antibodies against HLA Classes I and II

	Class I only	Class II only	Classes I and II	Test statistic	*p*
Age (years)	53 (38–62)	45.5 (7–76)	57.5 (13–67)	2.001	0.368^a^
Transplant duration (years)	4 ± 6.08	5.88 ± 3.61	5.36 ± 2.87	0.369	0.694^b^
ALP	327.4 (84.3–342.6)	136 (36–196)	136.05 (27–391.2)	2.955	0.228^a^
AST (UL)	21.4 (20.2–1776.2)	19.3 (8.4–42)	24.15 (11.9–71)	2.900	0.235^a^
ALT (UL)	41 (11.6–692.1)	13.3 (8–83)	33.7 (9.9–168)	2.115	0.347^a^
Total bilirubin (mg/dL)	4.72 ± 4.48	1.32 ± 0.76	0.82 ± 0.3	3.390	0.121^b^
Direct bilirubin (mg/dL)	0.88 (0.57–5.54)^*a*^	0.27 (0.1–1.29)^a.b^	0.19 (0.1–0.56)^b^	9.572	0.008^a^
Class I MFI	2,234 (2,072–4,431)^a^	599 (134–976)^b^	1,989 (1,024–4,498)^a^	23.559	<0.001^a^
Class II MFI	276 (249–365)^a^	2,145 (1,153–5,477)^b^	2,044.5 (1,100–7,310)^b^	8.218	0.016^a^
1-Month TAC (U/L)	7.71 ± 0.65	8.26 ± 2.42	8.85 ± 3.17	0.306	0.739^b^
3-Month TAC (U/L)	9.76 ± 1.2	8.65 ± 2.68	9.39 ± 2.7	0.420	0.661^b^
6-Month TAC (U/L)	9.29 ± 0.85	8.01 ± 1.99	8.94 ± 1.95	1.148	0.331^b^

No difference between groups with the same letter. Values are represented as mean ± standard deviation and median (range).

^a^Kruskal–Wallis *H* test.

^b^One-way ANOVA.

Abbreviations: ALP: alkaline phosphatase, AST: aspartate transaminase, ALT: alanine transaminase, MFI: mean fluorescence intensity, TAC: tacrolimus.

#### 3.1.5 Demographic and pathological findings according to the presence of post-transplant *de novo* DSAs

DSA class showed no significant relationship with categorical variables such as sex, donor type, rejection, and biopsy findings (*p* > 0.05) (Table 6).

**Table 6:** Comparison of characteristics according to the presence of donor-specific antibodies against HLA Classes I and II

Parameter	Class I only	Class II only	Classes I and II	Test statistic	*p*
Donor					
Living	2 (66.7)	15 (93.8)	12 (85.7)	2.264	0.273^a^
Deceased	1 (33.3)	1 (6.3)	2 (14.3)		
Sex					
Male	1 (33.3)	10 (62.5)	6 (42.9)	1.652	0.515^a^
Female	2 (66.7)	6 (37.5)	8 (57.1)		
Rejection status					
No	2 (66.7)	12 (75)	9 (64.3)	0.693	0.862^a^
Yes	1 (33.3)	4 (25)	5 (35.7)		
Portal inflammation severity					
1	1 (100)	2 (40)	1 (20)	4.677	1.000^a^
2	0 (0)	2 (40)	2 (40)		
3	0 (0)	0 (0)	1 (20)		
4	0 (0)	1 (20)	1 (20)		
Lobular inflammation					
K1		1 (33.3)	0 (0)	1.956	0.648^a^
K2		1 (33.3)	1 (25)		
K3		1 (33.3)	3 (75)		
Fibrosis					
2	0 (0)	1 (33.3)	3 (60)	6.137	0.644^a^
3	0 (0)	1 (33.3)	0 (0)		
4	0 (0)	0 (0)	1 (20)		
5	1 (100)	1 (33.3)	1 (20)		
Ductulitis					
No	3 (100)	16 (100)	13 (92.9)	—	—
Yes	0 (0)	0 (0)	1 (7.1)		
Venulitis of central vein					
No	3 (100)	16 (100)	14 (100)	—	—
Venulitis of portal vein					
No	3 (100)	16 (100)	13 (92.9)	—	—
Yes	0 (0)	0 (0)	1 (7.1)		
Bile duct loss					
No	3 (100)	16 (100)	13 (92.9)	—	—
Yes	0 (0)	0 (0)	1 (7.1)		
Hyperchromatosis					
No	2 (66.7)	14 (87.5)	12 (85.7)	1.374	0.604^a^
Yes	1 (33.3)	2 (12.5)	2 (14.3)		
Nucleolus					
No	2 (66.7)	14 (87.5)	12 (85.7)	1.374	0.604^a^
Yes	1 (33.3)	2 (12.5)	2 (14.3)		
Cell disorganization					
No	2 (66.7)	14 (87.5)	12 (85.7)	1.374	0.604^a^
Yes	1 (33.3)	2 (12.5)	2 (14.3)		
Pathological diagnosis					
Steatohepatitis	0 (0)	0 (0)	1 (33.3)		

Values are represented as *n* (%).

^a^Fisher–Freeman–Halton test.

The association between independent risk factors and rejection was examined by binary logistic regression analysis using univariate and multivariate binary logistic regression models. Tacrolimus values at post-transplant 3 months were found to be significantly associated with rejection in the univariate and multivariate models (*p* = 0.010 and *p* = 0.030, respectively). With each unit increase in tacrolimus at 3 months, the odds of rejection were 1.353 times higher according to the univariate model and 1.337 times higher according to the multivariate model. Other variables showed no statistically significant association with rejection (*p* > 0.05) (Table 7).

**Table 7:** Binary logistic regression analysis of the association between independent risk factors and rejection

	Rejection		Univariate		Multivariate	
	Negative	Positive	OR (95% CI)	*p*	OR (95% CI)	*p*
Transplant type						
Living donor	63 (75)	21 (25)	Reference			
Deceased donor	6 (60)	4 (40)	2 (0.514–7.778)	0.317	1.957 (0.334–11.481)	0.457
Diagnosis						
Alveolar echinococcosis	11 (84.6)	2 (15.4)	—	—	—	—
Viral hepatitis B	24 (75)	8 (25)	—	—	—	—
Hepatocellular carcinoma	10 (90.9)	1 (9.1)	—	—	—	—
Cryptogenic hepatitis	6 (42.9)	8 (57.1)	—	—	—	—
Autoimmune hepatitis	13 (68.4)	6 (31.6)	—	—	—	—
Wilson disease	5 (100)	0 (0)	—	—	—	—
Class I			—	—	—	—
Class I negative	58 (75.3)	19 (24.7)	Reference			
Class I positive	11 (64.7)	6 (35.3)	1.665 (0.542–5.111)	0.373	0.594 (0.028–12.408)	0.737
Class II						
Class II negative	48 (75)	16 (25)	Reference			
Class II positive	21 (70)	9 (30)	1.286 (0.49–3.372)	0.609	1.144 (0.151–8.671)	0.896
Sex						
Male	34 (70.8)	14 (29.2)	Reference			
Female	35 (76.1)	11 (23.9)	0.763 (0.304–1.915)	0.565	0.849 (0.26–2.771)	0.787
Age	45.54 ± 18.11	46.24 ± 13.75	1.002 (0.976–1.03)	0.858	0.998 (0.963–1.035)	0.927
ALP (U/L)	135.5 ± 76.59	158.05 ± 84.96	1.003 (0.998–1.009)	0.231	1 (0.992–1.008)	0.994
AST (U/L)	90.8 ± 348.09	238.85 ± 712.02	1.001 (1–1.001)	0.210	0.997 (0.988–1.005)	0.447
ALT (U/L)	84.21 ± 334.95	120.36 ± 274.55	1 (0.999–1.002)	0.639	1 (0.993–1.007)	0.994
Total bilirubin (mg/dL)	1.06 ± 0.88	2.49 ± 4.15	1.304 (0.998–1.704)	0.052	0.589 (0.093–3.711)	0.573
Direct bilirubin (mg/dL)	0.34 ± 0.53	1.29 ± 2.68	1.566 (0.985–2.489)	0.058	9.482 (0.408–220.581)	0.161
Class I MFI	725.01 ± 916.05	1070.54 ± 1208.97	1 (1–1.001)	0.158	1.001 (0.999–1.002)	0.329
Class II MFI	1094.68 ± 1404.4	1157.04 ± 1235.28	1 (1–1)	0.845	1 (0.999–1.001)	0.526
1-Month TAC (ng/mL)	7.8 ± 3.02	8.89 ± 2.2	1.139 (0.971–1.335)	0.110	1.1 (0.907–1.333)	0.333
3-Month TAC (ng/mL)	8.68 ± 2.1	10.09 ± 2.37	1.353 (1.075–1.702)	0.010	1.337 (1.029–1.737)	0.030
6-Month TAC (ng/mL)	8.46 ± 2.18	9.2 ± 1.82	1.187 (0.948–1.485)	0.135	1.121 (0.818–1.536)	0.477

Abbreviations: OR: odds ratio, CI: confidence interval, ALP: alkaline phosphatase, AST: aspartate transaminase, ALT: alanine transaminase, MFI: mean fluorescence intensity, TAC: tacrolimus.

## 4 Discussion

Our knowledge of DSAs in liver transplant recipients has evolved significantly over the past decade, and DSAs are no longer considered clinically insignificant to liver transplant outcomes.

### 4.1 Literature

In a 2012 study investigating the prevalence, development, and effects of anti-HLA DSAs in liver transplant patients, Taner et al. examined the frequency of DSAs at 1 year post-transplant and how these antibodies changed over time. They observed that DSAs were rare in the patient population and disappeared in most patients over time, noting that the presence of DSAs did not have a significant negative effect on transplant outcomes [15].

Vandevoorde et al. investigated the prevalence of DSAs after adult liver transplantation, the risk factors for their development, and the effects of these antibodies on transplantation outcomes. They determined that the presence of DSA can potentially lead to complications and unfavorable outcomes after liver transplantation but noted that these negative effects can vary from patient to patient [16].

In a 2013 study, O’Leary et al. reported that preformed Class II DSAs significantly increased the risk of rejection in the early post-transplant period [17]. Another 2013 study by Kaneku et al. revealed that *de novo* anti-HLA DSAs significantly reduced both patient and graft survival [18].

Del Bello et al. investigated how newly developed anti-HLA DSAs cause rejection reactions in liver transplant patients. Their findings indicated that the presence of *de novo* antibodies may trigger post-transplant rejection events, which can impact patients’ clinical course. The authors stated that *de novo* DSA monitoring is critical to increase post-transplant success [19].

In an earlier 2014 study, Del Bello et al. investigated the prevalence and incidence of DSA antibodies in post-liver transplant care patients and the risk factors associated with DSA. They found that DSAs are common and identified certain risk factors associated with antibody development [20].

Another study by O’Leary et al. examined the effects of post-transplant DSAs on the progression of liver fibrosis in liver transplant patients infected with the hepatitis C virus. They reported that DSAs can accelerate the development of fibrosis after liver transplantation and this may have negative effects on patients’ long-term health outcomes. The authors emphasized that anti-HLA antibodies are critically important, especially in transplant patients with hepatitis C infection [21].

A 2016 study by Ducreux et al. investigated the methods used to monitor the effectiveness of the treatment of humoral rejection after liver transplantation. The efficacy of Luminex single antigen tests and complement binding properties in this monitoring process was evaluated. The authors stated that Luminex tests may be useful, but the effectiveness of these tests should be confirmed by more clinical studies [22].

In another study examining DSA presence, risk factors, and long-term effects, preformed DSA was detected in 8 patients (24.2%) but titers of these antibodies were negative after 5 years and no adverse events occurred. Eight (33.3%) of the other 24 patients developed *de novo* DSA, but only 2 patients remained Class II DSA-positive with high MFI at 5 years. There was no significant relationship between DSA development and the risk of rejection, graft loss, or mortality [23].

In a study by Liu et al. examining the role of DSAs in graft survival after pediatric liver transplantation, DSA positivity was detected in 10 (20.8%) of 48 patients and AMR was observed in 4 patients. The authors concluded that DSA positivity was an independent risk factor for liver failure and long-term mortality [24].

Kovandova et al. revealed in their study that *de novo* HLA Class II antibodies were associated with chronic AMR but not acute AMR after liver transplantation [25].

A retrospective study evaluating the frequency, developmental risk factors, and effects of anti-HLA DSAs in adults after first liver transplantation between 2000 and 2010 showed that the presence of DSAs did not significantly impact patient or graft survival. DSAs were detected in a minority of adult liver transplant patients and were reported to generally have a limited impact on graft and patient outcomes [16].

A systematic review and meta-analysis by Beyzaei et al. examined the effects of *de novo* DSA on acute and chronic rejection rates and long-term graft survival. The results indicated that *de novo* DSA may increase the risk of graft rejection and loss [26].

The study by Beyzaei et al. focused on the effect of *de novo* DSAs on long-term liver transplant outcomes. Although it provided specific findings on the effects of *de novo* DSAs on clinical outcomes, data regarding MFI values were not included in the study.

In a 2019 study by Jucaud et al., *de novo* DSA development was detected in 69 liver transplant patients. Only adult and pediatric patients receiving calcineurin inhibitor monotherapy were included in the study. MFI values were considered positive above 1,000, but specific findings and details were not reported [27].

In their 2022 study, Pinon et al. found a high rate of *de novo* DSA positivity in pediatric liver transplant patients. The study revealed that *de novo* DSAs were common in these patients, potentially affecting long-term transplant outcomes. However, the study did not specifically include detailed reports on the clinical effects of *de novo* DSAs [28].

There is no widely accepted significant MFI threshold value associated with high risk for patients undergoing liver transplantation [27,29]. However, in small-scale studies using lower MFI threshold values, there was generally no association with clinically significant adverse events. Clinical scoring systems based on DSA and MFI have been suggested to improve the prediction of long-term liver allograft survival in patients with chronic AMR lesions. Additional molecular antibody and complement binding assays may aid in predicting liver allograft fibrosis and the risk of acute and chronic rejection. However, there are limited studies evaluating these tests in the context of liver allograft loss [20,30,31].

In most studies, regardless of being preformed or *de novo*, DSA positivity was associated with more allograft inflammation and fibrosis, but studies in which preformed DSAs became negative after transplantation did not confirm these relationships. HLA epitope mismatch scores may help identify liver patients at higher risk of developing *de novo* DSA, but the results are preliminary. As with other organ transplants, nonadherence to drug therapy and inadequate immunosuppression can significantly impact DSA incidence and risk.

It is also worth noting that bilirubin levels are an important marker in DSA-positive patients. DSA positivity should be evaluated as a clinically relevant biomarker and clinicians should be aware that elevated bilirubin levels can lead to liver damage and seriously threaten graft survival in DSA-positive transplant patients. Therefore, regular pathological examinations and periodic DSA tests during the follow-up of these patients may be critical in maintaining graft survival.

Furthermore, DSA positivity was observed to have no direct effect on immunosuppression therapy. The current literature suggests that higher MFI values (>1,000) are associated with adverse histological changes in the liver allograft. This fact may improve long-term survival prediction with clinical scoring systems.

In the present study, a statistically significant relationship between DSA positivity and risk of rejection or liver damage was not observed. This finding shows that DSA positivity is not directly associated with the expected negative outcomes in this patient group. However, there are reports in the literature suggesting that DSA should be considered as a potential biomarker in clinical management, so clinicians should keep in mind that this result may differ in specific patient subgroups.

## 5 Conclusion

This study evaluated the effects of DSA positivity on the graft in liver transplant patients with various liver diseases and showed that the impact of DSAs on liver damage and rejection was generally limited. Although DSA-positive patients were found to have high MIF values, especially those in diagnostic groups such as Wilson disease and autoimmune hepatitis, no significant association with rejection or liver damage was observed. In addition, DSAs persisting after the first year had no apparent effect on the liver. However, it is important to monitor DSA persistence and titers at certain periods in patients with early positivity after transplantation. It should be taken into account that elevated bilirubin levels in DSA-positive transplant patients may lead to liver damage and seriously threaten graft survival. Therefore, regular pathological examinations and periodic DSA tests during follow-up may be critical for graft survival in these patients. Overall, DSA positivity and immunosuppression therapy were not observed to have a statistically significant impact on rejection, and the results highlight the need for larger-scale studies to understand the effects of DSA on the course of liver transplantation.

### 5.1 Limitations

This research was a single-center cohort study with retrospective and prospective elements, and the results should be considered preliminary. It is important to recognize that this type of study design may introduce certain biases. In all patients except deceased donors, anti-HLA antibody detection was performed within an average of one year from transplantation, so our estimates of the course of antibody development over time are reliable. However, it is not possible to comment on the immunological status of patients in the following years. As lifestyle factors or treatment adherence during follow-up were not evaluated in our study, we also cannot comment on the effects of these variables on clinical outcomes and DSA formation. In subsequent studies, the individual characteristics, immunological profiles, and clinical conditions of the patients before and after transplantation will be analyzed in more depth. Characteristics such as DSA type, binding ability, titer, and complement binding capacity are planned to be characterized in detail. This will enable the determination of the most appropriate treatment approaches for each patient.

Future research should focus on the development of individualized treatment plans to prevent complications, increase survival rates, and improve the quality of life in patients who develop DSA.
